# Spectral-brightness optimization of an X-ray free-electron laser by machine-learning-based tuning

**DOI:** 10.1107/S1600577523007737

**Published:** 2023-10-27

**Authors:** Eito Iwai, Ichiro Inoue, Hirokazu Maesaka, Takahiro Inagaki, Makina Yabashi, Toru Hara, Hitoshi Tanaka

**Affiliations:** a Japan Synchrotron Radiation Research Institute, 1-1-1 Kouto, Sayo, Hyogo 679-5198, Japan; b RIKEN SPring-8 Center, 1-1-1 Kouto, Sayo, Hyogo 679-5148, Japan; RIKEN SPring-8 Center, Japan

**Keywords:** X-ray free-electron lasers, machine learning, beam tuning, SACLA, spectral-brightness optimization, single-shot inline spectrometers

## Abstract

A machine-learning-based beam optimizer has been implemented to maximize the spectral brightness of the X-ray free-electron laser (XFEL) pulses of SACLA. A new high-resolution single-shot inline spectrometer capable of resolving features of the order of a few electronvolts was employed to measure and evaluate XFEL pulse spectra. Compared with a simple pulse-energy-based optimization, the spectral width was narrowed by half and the spectral brightness was improved by a factor of 1.7. The optimizer significantly contributes to efficient machine tuning and improvement of XFEL performance at SACLA.

## Abstract 2

Spectral-brightness optimization was performed by using a machine-learning-based beam optimizer and a new high-resolution inline spectrometer.

## 1. Introduction

X-ray free-electron lasers (XFELs) based on a self-amplified spontaneous-emission scheme are X-ray sources that generate femtosecond hard X-ray laser pulses (Pellegrini *et al.*, 2016 ▸). In combination with their high transverse coherence and high photon flux, XFELs provide novel experimental opportunities, such as damage-free protein crystallography (Schlichting, 2015 ▸; Hirata *et al.*, 2014 ▸), coherent imaging of nanomaterials (Clark *et al.*, 2013 ▸; Kimura *et al.*, 2014 ▸; Yumoto *et al.*, 2022 ▸), time-resolved analysis of chemical reactions (Kim *et al.*, 2015 ▸, 2020 ▸; Katayama *et al.*, 2019 ▸), generation and diagnostics of materials with high-energy densities (Vinko *et al.*, 2012 ▸; Inoue *et al.*, 2016 ▸, 2021*a* ▸, 2022*a* ▸), and exploring non-linear X-ray optical phenomena (Glover *et al.*, 2012 ▸; Yoneda *et al.*, 2015 ▸; Tamasaku *et al.*, 2018 ▸; Inoue *et al.*, 2021*b* ▸).

The key photon parameters for performing these experiments are different from experiment to experiment. For example, spectral brightness is important in spectroscopic experiments to obtain high data throughputs. In diffraction experiments, pulse energy determines the data quality as long as diffraction peaks are not blurred by bandwidth broadening.

As described below, SACLA (Ishikawa *et al.*, 2012 ▸) has a variety of radiofrequency (RF) cavities in its injector section, and beam tuning is complicated and time consuming. This difficult beam tuning has so far relied on machine operators’ skills, but recent developments in machine-tuning techniques based on machine-learning algorithms can now greatly expand the speed and flexibility of machine tuning (Duris *et al.*, 2020 ▸).

We have implemented a machine-learning-based beam optimizer, which can be used not only for XFEL machines but also in other accelerator facilities, such as a hadron cyclotron. In this article, we apply this beam optimizer to directly maximize spectral brightness using a high-resolution inline spectrometer at SACLA.

## 2. XFEL facility: SACLA

### 2.1. SACLA linac

**Figure 1:**
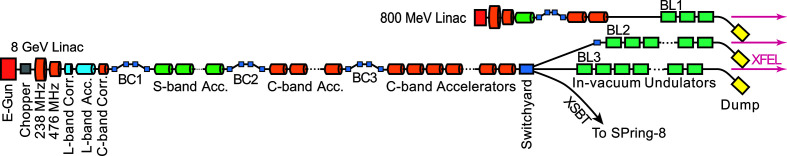
A schematic view of the SACLA accelerator.

As shown in Fig. 1 ▸, there are two linear accelerators (linacs) at SACLA: an 8 GeV main linac and an 800 MeV small linac. The 8 GeV linac is used for two hard XFEL beamlines (BL2 and BL3), and also beam injection to the SPring-8 storage ring (XSBT) started in 2020. The 800 MeV linac provides electron beams for a soft X-ray FEL beamline (BL1).

Since SACLA employs a thermionic electron gun (Togawa *et al.*, 2007 ▸) and velocity bunching in an injector section, the initial electron bunch has a relatively low current of 1 A and a long duration of 1 ns compared with an RF photo-cathode gun system. The electron beam is then longitudinally compressed to 10 fs with a peak current of 10 kA by three bunch compressors (Togawa *et al.*, 2009 ▸). The frequencies of RF acceleration fields are increased proportionally to the bunch length, from 238 MHz, 476 MHz, L band (1428 MHz), S band (2856 MHz) to C band (5712 MHz) (Inagaki *et al.*, 2014 ▸). To compensate for non-linearity due to the RF curvatures and bunch compressors, L-band and C-band correction cavities are installed before the first bunch compressor. In transverse planes, magnetic solenoid lenses are used to focus the electron beam in the low-energy injector section and quadrupole magnets are used after the L-band accelerator. The bunch repetition rate is 60 Hz and the accelerated bunches are distributed in three directions (BL2, BL3 and XSBT) using a kicker magnet (Hara *et al.*, 2016 ▸). In-vacuum undulators (Kitamura, 2000 ▸) with a 18 mm period are used for FEL generation. Each undulator segment has a length of 5 m and there are 3, 17 and 19 segments installed for BL1, BL2 and BL3, respectively.

### 2.2. Tuning knobs

XFELs require a high-density electron beam in six-dimensional phase space. Thus, the electron bunch should be properly compressed in a longitudinal direction with minimum degradation of transverse emittance. It is also important to match the transverse beam envelope to periodic beam focusing optics of the undulator section. To ensure spatial and spectral overlap between the electron bunch and the XFEL photon pulse, undulator *K* values, taper and electron-beam orbit should be properly set in the undulator section.

The tuning knobs to adjust these parameters are summarized in Table 1 ▸. The longitudinal bunch compression is driven by an energy chirp applied by the off-crest acceleration, so the bunch length and peak current are optimized by the seven RF phases of 238 MHz cavity, 476 MHz cavity, L-band corrector (LBC), L-band accelerator (LB), C-band corrector (CBC), S-band accelerator (SB) and C-band accelerator (CB1-3, before the third bunch compressor). The transverse beam envelope and orbit are adjusted by the magnetic lenses, quadrupole magnets and steering magnets, as listed in Table 1 ▸. Since the beam energy is gradually reduced in the undulator section due to the FEL interaction and wakefields, the undulator *K* values and its taper are also tuned by the beam optimizer.

To increase the spectral brightness, the spectral bandwidth should be reduced while keeping the pulse intensity the same. The XFEL spectral bandwidth is considered to be mainly sensitive to the RF phases and undulator gaps. Since collective effects, such as coherent synchrotron radiation and wakefields, change the electron-energy chirp and affect the XFEL spectrum, the transverse optics of the electron beam are also adjusted to minimize these effects.

Because of a variety of RF cavities and a complicated injector section, there are much more tuning knobs in SACLA compared with an RF photo-cathode-based linac. On top of that, the parameters of the electron bunch, such as energy, length and peak current, should be optimized individually for three destinations.

Recent detailed demands from XFEL users, such as optimization of spectral brightness and transverse profiles, make the introduction of a machine-learning-based beam optimizer inevitable to improve the efficiency of the beam-tuning process.

### 2.3. Spectrometer

In SACLA, a double-crystal monochromator is used to measure an averaged spectrum with a relative energy resolution (normalized by the central photon energy) of Δ*E*/*E* ≃ 1 × 10^−4^ eV and an inline spectrometer (Tono *et al.*, 2013 ▸) (ISpec) is used to monitor a single-shot spectrum. The ISpec detects Bragg diffraction of a thin polycrystalline diamond film and its resolution is ∼100 eV full width at half-maximum (FWHM) at 10 keV, which is larger than a typical XFEL bandwidth of 40 eV FWHM at 10 keV.

**Figure 2:**
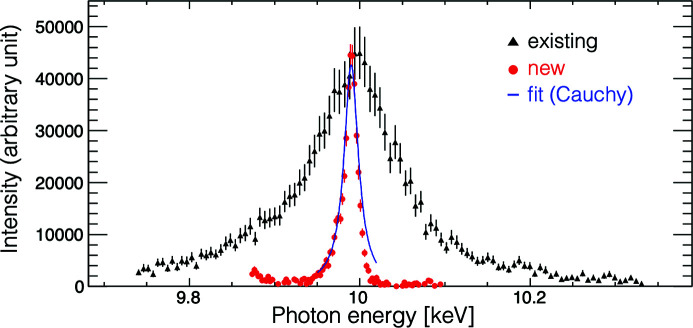
An example of a spectrum obtained with the existing spectrometer (black triangles) and the new spectrometer (red circles). The obtained spectra are fitted with the Cauchy distribution to evaluate spectral bandwidth (blue line).

To measure and optimize the spectral brightness of the XFEL on a single-shot basis, a high-resolution ISpec was newly introduced (Inoue *et al.*, 2022*b* ▸). Instead of a diamond film, the new ISpec employs a 0.3 mm-diameter capillary filled with 3 µm-diameter diamond microcrystals. The capillary is transversely inserted into a halo of XFEL photon pulses, and a spectral resolution of a few electronvolts can be obtained without any complicated alignments. The spectra measured by the old and new ISpecs are compared in Fig. 2 ▸. An XFEL spectral width of 25 eV FWHM was correctly obtained with the new ISpec and it is used for the spectral-brightness optimization of XFEL photon pulses.

## 3. Optimizer

A new beam optimizer is implemented based on Bayesian optimization with Gaussian process regression (GPR) (Rasmussen & Williams, 2006 ▸), one of the machine-learning methods. Hereafter, we refer to it as BO.

### 3.1. Gaussian process regression

GPR is a type of Bayesian regression where the probability distribution of each parameter is supposed to be Gaussian. We consider a regression model with Gaussian noise,

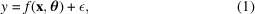

where **x** is an input vector, *y* is an output value, **θ** is a hyperparameter vector and ε is Gaussian noise. In the case of XFELs, for example, **x** is a tuning parameter set of the accelerator, *y* is the XFEL intensity and ε is the intrinsic fluctuation of XFEL intensity. After taking *N* data samples, 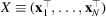 and **y** ≡ 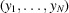 , we can estimate the output y* at an arbitrary input vector x* from the data by GPR. From the Bayes theorem, we get 

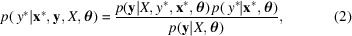

where *p*(y*|x*, **θ**) is the prior distribution of the parameters, *p*(**y**|*X*, y*, x*, **θ**) is the conditional probability to obtain the outputs **y** at the inputs *X* from the model function *f*, *p*(y*|x*, **y**, *X*, **θ**) is the posterior distribution after taking the data and *p*(**y**|*X*, **θ**) is the marginal likelihood of **y** occurring at *X*.

In GPR, the posterior probability of a test observation, y* at x*, is Gaussian, where the contribution to the posterior probability is inversely proportional to the squared distance between the inputs of the test and each data point |x* − **x**_*n*_|^2^ if we take a radial basis function (RBF) for the kernel. Here, the squared distance is calculated using the length-scale hyperparameter θ_*k*_, 

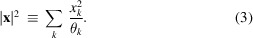

The shape of the function *f* is determined by the kernel function, observed data and the prior distribution. The hyperparameters of the model are thus the length scale for the distance calculation, the variance of the measurement noise, and the mean and variance of the prior distribution. The hyperparameters are updated after the observation of a new data sample so that the marginal likelihood is maximized. Here, the marginal likelihood is defined as 

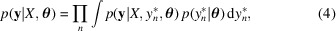

where 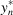 is defined as y* at the input vector x* = **x**_*n*_. In this way, the probability distribution of the model function *f* is updated by taking a new data sample.

For an optimization problem, the next sampling point could be intelligently selected by utilizing the expected improvement (Garnett, 2023 ▸) (EI), which is defined as an expected marginal gain compared with the best point so far,

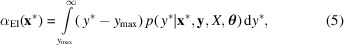

where *y*_max_ is the best output value observed so far. In the BO, the next observation point is set to the maximum point of the EI for each step.

### 3.2. Optimizer software

The BO (Iwai *et al.*, 2021 ▸) was implemented based on *scikit-learn* (Pedregosa *et al.*, 2011 ▸) and *GPyTorch* (Gardner *et al.*, 2018 ▸)/*BoTorch* (Balandat *et al.*, 2020 ▸). The GPR kernel, *K*, is defined based on the RBF as 

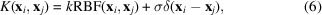

where *k* and σ are some coefficient and noise hyperparameters, respectively (this can be represented by the convolution of the ‘ConstantKernel’, ‘RBF’ and ‘WhiteKernel’ with *scikit-learn*). With this model, hyperparameters are thus length scale, noise level, mean and variance of the prior distribution, as mentioned in Section 3.1. The given parameter ranges are normalized, treated equally in the BO, and the length scale for each parameter is limited to the range [0.075, 1.0]. The length scale is a characteristic length of ‘structures’ in a given parameter space and the inverse number of the length scale corresponds to the number of structures to be considered in a given parameter space, because the given parameter ranges are normalized. Thus a length scale of 1 may give a smooth curve without any structures, and a small number of the length scale may give some detailed structures, but sometimes overfitting certain samples. The specified limits on length scale roughly mean just considering 1–10 structures in each parameter range, and also prevent the optimization process from overfitting certain samples. As for noise level, its initial value and range are set according to the obtained data. At the beginning of the optimization process, we obtain data, calculate the given performance index for each and evaluate the fluctuation σ_obs_, and the noise-level bounds are set to [σ_obs_/3, σ_obs_]. For pulse-energy-related maximized optimization, the prior distribution is set to 0. After taking new sample data, the GPR model is updated to maximize the marginal likelihood. The next parameters are then derived as they give the largest EI.

**Figure 3:**
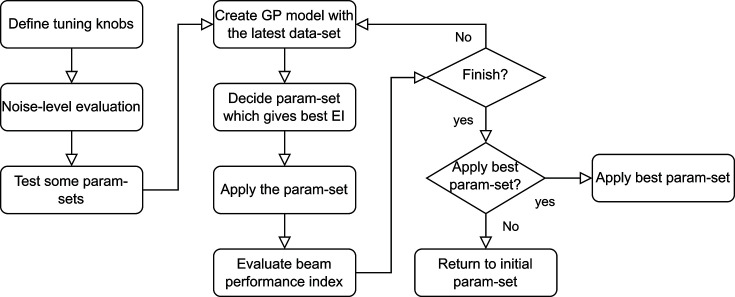
A flowchart of the BO.

**Figure 4:**
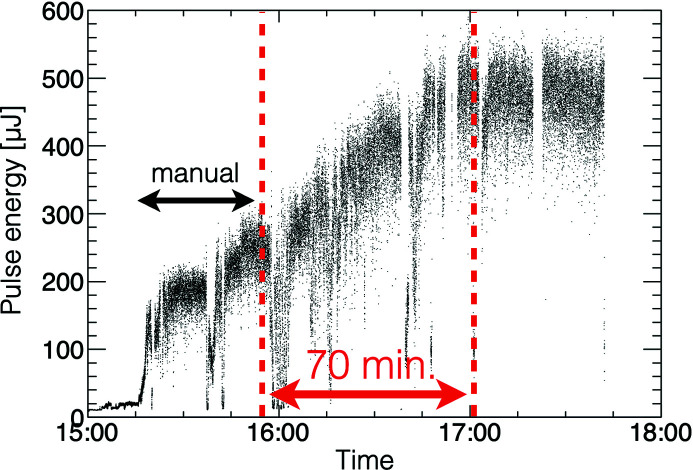
An example of XFEL pulse-energy optimization using the BO.

The BO has been implemented so that even inexperienced operators can use it from the graphical user interface, and today it is commonly used for daily accelerator tuning. After simply specifying a template of tuning knobs, the optimization process can be started. The BO is able to handle up to 32 knobs simultaneously but it is currently limited to 16 parameters to finish the optimization process within 30 mins to an hour. With the specified tuning knobs, the optimization process starts with the noise-level evaluation as shown in Fig. 3 ▸. It usually acquires 300 samples, 10 s at 30 Hz, to evaluate noise level. Then, the BO tests some parameter sets, depending on their initial values and given parameter ranges. For example, when the initial value is placed in the middle (50%) of the parameter range, 1/4 from both ends, 25% and 75%, are tested for each parameter. Based on the first data set, a GPR model is created and a parameter set, which gives the best EI, is calculated. After applying the parameter set, the BO waits 0.5 s for RF phases and 3.0 s for quadrupole magnets depending on the response time of devices (minimum: 0.5 s). Ninety pulses, 3 s at 30 Hz, are used for the evaluation of the latest parameter set, and the GPR model is then updated. Applying the control parameters, evaluating the XFEL and updating the GPR model constitute one cycle. This cycle is usually repeated up to 100 cycles, and the optimization process takes 15–30 min depending on the number of tuning knobs. The operator can terminate the optimization at any time, either by resetting the parameters to their initial values or applying the best parameters at that moment. Fig. 4 ▸ shows an example of XFEL pulse-energy optimization using the BO.

## 4. Spectral-brightness optimization

In the spectral-brightness optimization, the new ISpec was used to maximize the spectral brightness, *B*_avg_, defined as 

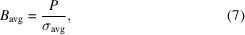

where

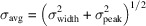

and

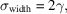

where *P* is the XFEL pulse energy, σ_width_ denotes the spectral bandwidth and is defined as the FWHM of each spectrum, and γ is the scale parameter of the Cauchy distribution (the half width at half-maximum). The peak photon energy and the spectral bandwidth for each pulse were obtained by fitting the XFEL spectrum of the new ISpec to the Cauchy distribution, as shown in Fig. 2 ▸. σ_peak_ is the FWHM of the distribution of observed peak photon energies of 90 pulses in a cycle; it is introduced to penalize broadening of the average spectrum due to the fluctuation of the peak photon energy.

**Figure 5:**
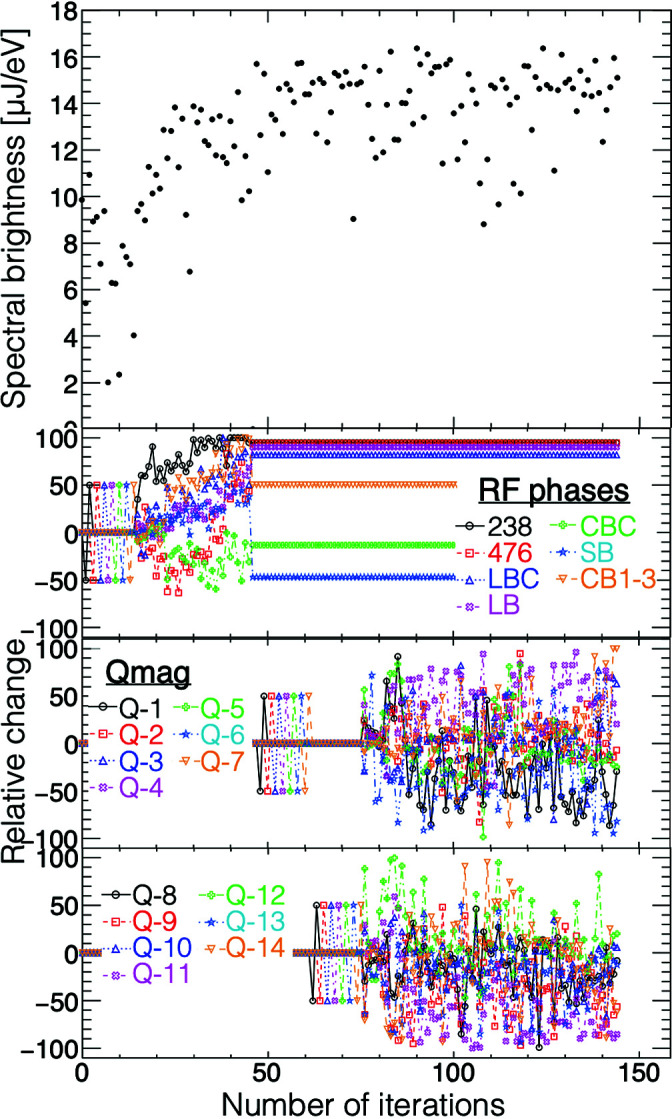
An example of the progress of the spectral-brightness optimization with the performance index defined by equation (7). The optimization began with the seven RF phases and then 14 quadrupole magnets, before the undulators were used as tuning knobs.

**Figure 6:**
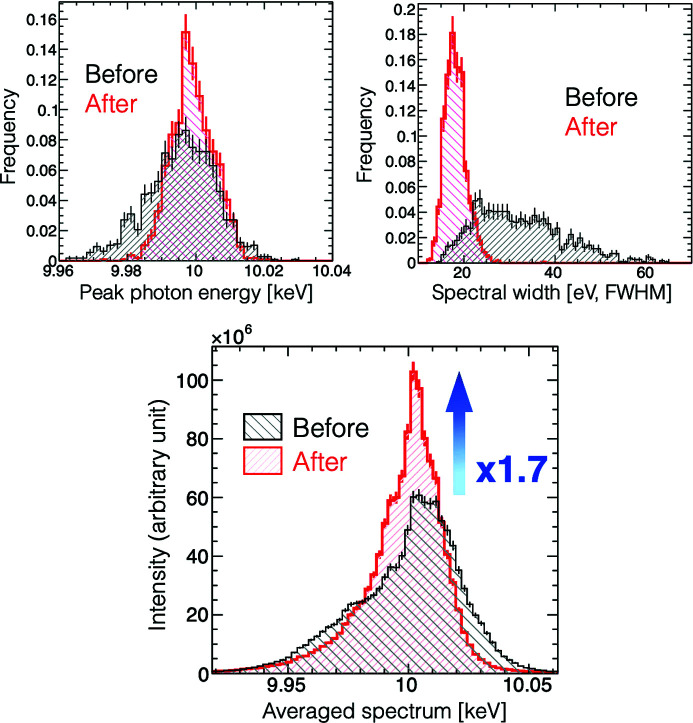
The distributions of the peak photon energy (top left), the single-shot spectral bandwidth (top right) and the averaged spectra, before (black) and after (red) the spectral-brightness optimization by the BO.

The BO was tested with the spectral brightness of equation (7) as a performance index. First, the pulse energy was simply maximized, and then the BO was used to further improve the spectral brightness. The optimization began with seven RF phases and then 14 quadrupole magnets, before the undulators were used as tuning knobs. The BO increased the performance index, *B*_avg_, by a factor of 1.7. The XFEL pulse energy was maintained at a similar level before/after/during the optimization process. Fig. 5 ▸ shows the progress of the spectral-brightness optimization. As shown in Fig. 6 ▸, the FWHM of the peak photon energy was improved from 23.8 to 14.8 eV, the mean value of the single-shot spectral bandwidth (FWHM) was improved from 32.2 to 18.2 eV and the spectral brightness in averaged spectra was certainly improved by a factor of 1.7.

## 5. Conclusions

We have implemented a beam optimizer based on Bayesian optimization to adjust the machine parameters of an XFEL automatically. When the XFEL pulse energy is chosen as a performance index, the optimizer sometimes does not give the best spectral brightness. To enable spectral-brightness optimization, a high-resolution single-shot inline spectrometer has been introduced. After searching for a performance index that appropriately represents the spectral brightness, the optimizer successfully improved the spectral brightness by a factor of 1.7 and the bandwidth was reduced by half. At SACLA, the beam optimizer significantly contributes to efficient machine tuning and improvement of XFEL performance.

## Figures and Tables

**Table 1:** Tuning knobs of SACLA to be adjusted by the BO

Parameter type	Number of parameters
RF phases	7 phases
Magnetic lens currents	9 units
Quadrupole-magnet currents	62 units
Steering-magnet currents	8 units
Undulator gaps (*K* values)	7

## References

[bb1] Balandat, M., Karrer, B., Jiang, D., Daulton, S., Letham, B., Wilson, A. G. & Bakshy, E. (2020). *Advances in Neural Information Processing Systems 33: Annual Conference on Neural Information Processing Systems 2020 (NeurIPS 2020)*, 6–12 December 2020, virtual. (https://doi.org/10.48550/arXiv.1910.06403.)

[bb2] Clark, J. N., Beitra, L., Xiong, G., Higginbotham, A., Fritz, D. M., Lemke, H. T., Zhu, D., Chollet, M., Williams, G. J., Messerschmidt, M., Abbey, B., Harder, R. J., Korsunsky, A. M., Wark, J. S. & Robinson, I. K. (2013). *Science*, **341**, 56–59. 10.1126/science.1236034 23704372

[bb3] Duris, J., Kennedy, D., Hanuka, A., Shtalenkova, J., Edelen, A., Baxevanis, P., Egger, A., Cope, T., McIntire, M., Ermon, S. & Ratner, D. (2020). *Phys. Rev. Lett.* **124**, 124801. 10.1103/PhysRevLett.124.124801 32281869

[bb4] Gardner, J. R., Pleiss, G., Bindel, D., Weinberger, K. Q. & Wilson, A. G. (2018). *arXiv*:1809.11165.

[bb5] Garnett, R. (2023). *Bayesian Optimization*, ch. 7. Cambridge University Press.

[bb6] Glover, T. E., Fritz, D. M., Cammarata, M., Allison, T. K., Coh, S., Feldkamp, J. M., Lemke, H., Zhu, D., Feng, Y., Coffee, R. N., Fuchs, M., Ghimire, S., Chen, J., Shwartz, S., Reis, D. A., Harris, S. E. & Hastings, J. B. (2012). *Nature*, **488**, 603–608. 10.1038/nature11340 22932384

[bb7] Hara, T., Fukami, K., Inagaki, T., Kawaguchi, H., Kinjo, R., Kondo, C., Otake, Y., Tajiri, Y., Takebe, H., Togawa, K., Yoshino, T., Tanaka, H. & Ishikawa, T. (2016). *Phys. Rev. Accel. Beams*, **19**, 020703.

[bb8] Hirata, K., Shinzawa-Itoh, K., Yano, N., Takemura, S., Kato, K., Hatanaka, M., Muramoto, K., Kawahara, T., Tsukihara, T., Yamashita, E., Tono, K., Ueno, G., Hikima, T., Murakami, H., Inubushi, Y., Yabashi, M., Ishikawa, T., Yamamoto, M., Ogura, T., Sugimoto, H., Shen, J. R., Yoshikawa, S. & Ago, H. (2014). *Nat. Methods*, **11**, 734–736. 10.1038/nmeth.2962 24813624

[bb9] Inagaki, T., Kondo, C., Maesaka, H., Ohshima, T., Otake, Y., Sakurai, T., Shirasawa, K. & Shintake, T. (2014). *Phys. Rev. ST Accel. Beams*, **17**, 080702.

[bb11] Inoue, I., Deguchi, Y., Ziaja, B., Osaka, T., Abdullah, M. M., Jurek, Z., Medvedev, N., Tkachenko, V., Inubushi, Y., Kasai, H., Tamasaku, K., Hara, T., Nishibori, E. & Yabashi, M. (2021*a*). *Phys. Rev. Lett.* **126**, 117403. 10.1103/PhysRevLett.126.117403 33798368

[bb12] Inoue, I., Inubushi, Y., Osaka, T., Yamada, J., Tamasaku, K., Yoneda, H. & Yabashi, M. (2021*b*). *Phys. Rev. Lett.* **127**, 163903. 10.1103/PhysRevLett.127.163903 34723578

[bb10] Inoue, I., Inubushi, Y., Sato, T., Tono, K., Katayama, T., Kameshima, T., Ogawa, K., Togashi, T., Owada, S., Amemiya, Y., Tanaka, T., Hara, T. & Yabashi, M. (2016). *Proc. Natl Acad. Sci. USA*, **113**, 1492–1497. 10.1073/pnas.1516426113 PMC4760814 26811449

[bb14] Inoue, I., Iwai, E., Hara, T., Inubushi, Y., Tono, K. & Yabashi, M. (2022*b*). *J. Synchrotron Rad.* **29**, 862–865. 10.1107/S1600577522001205 PMC9070727 35511018

[bb13] Inoue, I., Tkachenko, V., Kapcia, K. J., Lipp, V., Ziaja, B., Inubushi, Y., Hara, T., Yabashi, M. & Nishibori, E. (2022*a*). *Phys. Rev. Lett.* **128**, 223203. 10.1103/PhysRevLett.128.223203 35714226

[bb15] Ishikawa, T., Aoyagi, H., Asaka, T., Asano, Y., Azumi, N., Bizen, T., Ego, H., Fukami, K., Fukui, T., Furukawa, Y., Goto, S., Hanaki, H., Hara, T., Hasegawa, T., Hatsui, T., Higashiya, A., Hirono, T., Hosoda, N., Ishii, M., Inagaki, T., Inubushi, Y., Itoga, T., Joti, Y., Kago, M., Kameshima, T., Kimura, H., Kirihara, Y., Kiyomichi, A., Kobayashi, T., Kondo, C., Kudo, T., Maesaka, H., Maréchal, X. M., Masuda, T., Matsubara, S., Matsumoto, T., Matsushita, T., Matsui, S., Nagasono, M., Nariyama, N., Ohashi, H., Ohata, T., Ohshima, T., Ono, S., Otake, Y., Saji, C., Sakurai, T., Sato, T., Sawada, K., Seike, T., Shirasawa, K., Sugimoto, T., Suzuki, S., Takahashi, S., Takebe, H., Takeshita, K., Tamasaku, K., Tanaka, H., Tanaka, R., Tanaka, T., Togashi, T., Togawa, K., Tokuhisa, A., Tomizawa, H., Tono, K., Wu, S., Yabashi, M., Yamaga, M., Yamashita, A., Yanagida, K., Zhang, C., Shintake, T., Kitamura, H. & Kumagai, N. (2012). *Nat. Photon.* **6**, 540–544.

[bb16] Iwai, E., Sugimoto, T., Joti, Y., Kubota, K., Tajiri, Y., Maesaka, H., Inagaki, T., Hara, T. & Tanaka, H. (2021). *Proceedings of the 18th Annual Meeting of Particle Accelerator Society of Japan*, 9–12 August 2021, Online, pp. 151–155. WEOB02.

[bb17] Katayama, T., Northey, T., Gawelda, W., Milne, C. J., Vankó, G., Lima, F. A., Bohinc, R., Németh, Z., Nozawa, S., Sato, T., Khakhulin, D., Szlachetko, J., Togashi, T., Owada, S., Adachi, S. I., Bressler, C., Yabashi, M. & Penfold, T. J. (2019). *Nat. Commun.* **10**, 3606. 10.1038/s41467-019-11499-w PMC6689108 31399565

[bb18] Kim, J. G., Nozawa, S., Kim, H., Choi, E. H., Sato, T., Kim, T. W., Kim, K. H., Ki, H., Kim, J., Choi, M., Lee, Y., Heo, J., Oang, K. Y., Ichiyanagi, K., Fukaya, R., Lee, J. H., Park, J., Eom, I., Chun, S. H., Kim, S., Kim, M., Katayama, T., Togashi, T., Owada, S., Yabashi, M., Lee, S. J., Lee, S., Ahn, C. W., Ahn, D. S., Moon, J., Choi, S., Kim, J., Joo, T., Kim, J., Adachi, S. I. & Ihee, H. (2020). *Nature*, **582**, 520–524.

[bb19] Kim, K. H., Kim, J. G., Nozawa, S., Sato, T., Oang, K. Y., Kim, T. W., Ki, H., Jo, J., Park, S., Song, C., Sato, T., Ogawa, K., Togashi, T., Tono, K., Yabashi, M., Ishikawa, T., Kim, J., Ryoo, R., Kim, J., Ihee, H. & Adachi, S. (2015). *Nature*, **518**, 385–389. 10.1038/nature14163 25693570

[bb21] Kimura, T., Joti, Y., Shibuya, A., Song, C., Kim, S., Tono, K., Yabashi, M., Tamakoshi, M., Moriya, T., Oshima, T., Ishikawa, T., Bessho, Y. & Nishino, Y. (2014). *Nat. Commun.* **5**, 3052. 10.1038/ncomms4052 PMC3896756 24394916

[bb20] Kitamura, H. (2000). *J. Synchrotron Rad.* **7**, 121–130. 10.1107/S0909049500002983 16609185

[bb22] Pedregosa, F., Varoquaux, G., Gramfort, A., Michel, V., Thirion, B., Grisel, O., Blondel, M., Prettenhofer, P., Weiss, R., Dubourg, V., Vanderplas, J., Passos, A., Cournapeau, D., Brucher, M., Perrot, M. & Duchesnay, É. (2011). *J. Mach. Learn. Res.* **12**, 2825–2830.

[bb23] Pellegrini, C., Marinelli, A. & Reiche, S. (2016). *Rev. Mod. Phys.* **88**, 015006.

[bb24] Rasmussen, C. E. & Williams, C. K. I. (2006). *Gaussian Process for Machine Learning.* MIT Press.

[bb25] Schlichting, I. (2015). *IUCrJ*, **2**, 246–255. 10.1107/S205225251402702X PMC4392417 25866661

[bb26] Tamasaku, K., Shigemasa, E., Inubushi, Y., Inoue, I., Osaka, T., Katayama, T., Yabashi, M., Koide, A., Yokoyama, T. & Ishikawa, T. (2018). *Phys. Rev. Lett.* **121**, 083901. 10.1103/PhysRevLett.121.083901 30192600

[bb27] Togawa, K., Hara, T. & Tanaka, H. (2009). *Phys. Rev. ST Accel. Beams*, **12**, 080706.

[bb28] Togawa, K., Shintake, T., Inagaki, T., Onoe, K., Tanaka, T., Baba, H. & Matsumoto, H. (2007). *Phys. Rev. ST Accel. Beams*, **10**, 020703.

[bb29] Tono, K., Togashi, T., Inubushi, Y., Sato, T., Katayama, T., Ogawa, K., Ohashi, H., Kimura, H., Takahashi, S., Takeshita, K., Tomizawa, H., Goto, S., Ishikawa, T. & Yabashi, M. (2013). *New J. Phys.* **15**, 083035.

[bb30] Vinko, S. M., Ciricosta, O., Cho, B. I., Engelhorn, K., Chung, H., Brown, C. R. D., Burian, T., Chalupský, J., Falcone, R. W., Graves, C., Hájková, V., Higginbotham, A., Juha, L., Krzywinski, J., Lee, H. J., Messerschmidt, M., Murphy, C. D., Ping, Y., Scherz, A., Schlotter, W., Toleikis, S., Turner, J. J., Vysin, L., Wang, T., Wu, B., Zastrau, U., Zhu, D., Lee, R. W., Heimann, P. A., Nagler, B. & Wark, J. S. (2012). *Nature*, **482**, 59–62. 10.1038/nature10746 22278059

[bb31] Yoneda, H., Inubushi, Y., Nagamine, K., Michine, Y., Ohashi, H., Yumoto, H., Yamauchi, K., Mimura, H., Kitamura, H., Katayama, T., Ishikawa, T. & Yabashi, M. (2015). *Nature*, **524**, 446–449. 10.1038/nature14894 26310765

[bb32] Yumoto, H., Koyama, T., Suzuki, A., Joti, Y., Niida, Y., Tono, K., Bessho, Y., Yabashi, M., Nishino, Y. & Ohashi, H. (2022). *Nat. Commun.* **13**, 5300. 10.1038/s41467-022-33014-4 PMC9470745 36100607

